# Effect of Open-Wedge High Tibial Osteotomy and Lateral Retinacular Release on the Articular Cartilage of the Patellofemoral Joint: Analysis Using Magnetic Resonance Imaging T2 Mapping

**DOI:** 10.3390/jcm14020595

**Published:** 2025-01-17

**Authors:** Shuji Nakagawa, Hiroyuki Kan, Yuji Arai, Shintaro Komaki, Manabu Hino, Atsuo Inoue, Kenji Takahashi

**Affiliations:** 1 Department of Sports and Para-Sports Medicine, Graduate School of Medical Science, Kyoto Prefectural University of Medicine, Kawaramachi-Hirokoji, Kamigyo-ku, Kyoto 602-8566, Japan; shushi@koto.kpu-m.ac.jp; 2 Department of Orthopaedics, Kyoto Interdisciplinary Institute of Community Medicine, Mibu-Higashitakadacho, Nakagyo-ku, Kyoto 604-8845, Japan; kan180@jt6.so-net.ne.jp (H.K.); megane1031@gmail.com (M.H.); 3 Department of Orthopaedics, Graduate School of Medical Science, Kyoto Prefectural University of Medicine, Kawaramachi-Hirokoji, Kamigyo-ku, Kyoto 602-8566, Japan; s-komaki@koto.kpu-m.ac.jp (S.K.); a-inoue@koto.kpu-m.ac.jp (A.I.); t-keji@mbox.kyoto-inet.or.jp (K.T.)

**Keywords:** open wedge high tibial osteotomy, lateral retinacular release, patellofemoral joint, magnetic resonance imaging, cartilage

## Abstract

**Background/Objectives:** After open-wedge high tibial osteotomy (OWHTO), the patella is displaced distally, causing patellofemoral joint degeneration. The objective of this study was to ascertain whether the combination of OWHTO and lateral retinacular release (LRR) can prevent articular cartilage degeneration of the patellofemoral joint using magnetic resonance imaging T2 mapping. **Methods:** This study included 37 patients (37 knees) who underwent OWHTO alone (OWHTO group) and 37 patients (37 knees) who underwent OWHTO with LRR (OWHTO + LRR group) with a correction angle of <10° for varus knee osteoarthritis. MRI was performed on all knees prior to and 6 months after surgery to assess the patellar cartilage in sagittal images for T2 mapping. Three regions of interest, (the medial facet, patellar ridge, and lateral facet), were established for the articular cartilage on the patellar side. The T2 values were subsequently quantified. Lower limb alignment, patellar height, patellar tilt angle, and lateral shift ratio were evaluated pre-and post-surgery. **Results**: Mean T2 values at 6 months post-surgery of the medial facet and patellar ridge of the OWHTO group showed a significant increase after surgery; no significant changes were observed in either region in the OWHTO + LRR group. In both groups, a significant decrease in patellar tilt angle was observed postoperatively; no change was noted in the lateral shift ratio or congruence angle. The change in patellar tilt angle was significantly lower in the OWHTO + LRR group than in the OWHTO group. **Conclusions:** LRR combined with OWHTO prevented patellofemoral joint cartilage degeneration after surgery in cases of varus knee osteoarthritis.

## 1. Introduction

Osteoarthritis of the knee (KOA) is a degenerative disease primarily caused by deterioration of the articular cartilage. The main symptoms of OA are pain and loss of motion or function of the affected joint. Hyaline cartilage has a limited ability to regenerate due to its avascularity, lack of nerve endings, and very slow metabolism. KOA development is influenced by several factors, including occupation, sports participation, musculoskeletal injuries, gender, obesity, and malalignment of the lower limbs, such as genu varus or valgus [1]. Excessive loading of the articular cartilage also plays a role in disease progression. Medial open-wedge high tibial osteotomy (OWHTO) is a surgical procedure primarily used to correct genu varus alignment and is commonly used to preserve the joint in patients with KOA and reduce spontaneous knee osteonecrosis. Long-term results are stable, and in cases where the endpoint is total knee arthroplasty, a survival rate of >90% at 10 years after surgery has been reported [2]. Nevertheless, excessive correction in OWHTO can result in complications such as lateral hinge fractures, delayed bone union, and patellofemoral (PF) joint disorders, ultimately leading to unfavorable outcomes [3,4]. Goshima et al. reported that large gaps of >13 mm increase the risk of delayed bone union in OWHTO. Otakara et al. reported that a correction of >10° increases the risk of PF joint disorders [5]. Tanaka et al. reported that cartilage damage to the PF joint occurs when the opening gaps is >13 mm or the correction angle is >9° [6]. Therefore, inherent limitations exist regarding the extent of deformity correction that can be achieved using OWHTO. However, Komaki et al. reported that degeneration of the PF articular cartilage occurred even in cases where OWHTO correction was <10° on MRI [7]. Biplanar osteotomy is a useful tibial osteotomy technique; in addition to the transverse osteotomy of the posterior tibia, a second ascending osteotomy in the coronary plane beneath the tibial tuberosity is performed. This provides improved rotational stability of the osteotomy and creates an anterior buttress against sagittal tilting of the osteotomy planes [8]. As a result of the osteotomy performed at the proximal tibial tubercle to open the osteotomy site, the tibial tubercle is displaced in a distal direction relative to the osteotomy site. Increased pressure on the PF joint is a likely consequence with the potential for development of PF joint disorders. Therefore, prevention of an increase in pressure on the PF joint during OWHTO may be an effective method of preventing PF joint degeneration.

Conversely, pressure on the PF joint is reportedly reduced by lateral retinacular release (LRR). The indications for LRR alone are limited and the results remain undetermined [9,10]. However, it is a simple technique frequently used in combination with osteotomy around the knee. We postulated that the combination of OWHTO and LRR may be an effective method for preventing postoperative damage to PF joints. The magnetic resonance imaging (MRI) T2 mapping method is regarded as a valuable imaging evaluation technique for detecting early articular cartilage degeneration [7,11]. This method can elucidate qualitative alterations in articular cartilage by assessing modifications in the collagen organization and water content of the cartilage.

The objective of this study was to ascertain whether the combination of OWHTO and LRR could suppress degeneration of the articular cartilage of the PF joint using the MRI T2 mapping method. 

## 2. Materials and Methods

The protocol of this retrospective observational study was approved by the Institutional Review Board of our hospital (approval no. ERB-24-04). It was performed in accordance with the principles of the Declaration of Helsinki and its subsequent amendments or comparable ethical standards.

### 2.1. Study Cohort

A total of 74 knees that underwent OWHTO (*n* = 74; 23 male and 51 female) at our hospital between November 2017 and April 2023 were enrolled in this study. From November 2017 to October 2019, OWHTO was performed as a standalone procedure (OWHTO group). From November 2019 to April 2023, OWHTO was performed in combination with LRR (OWHTO + LRR group). The inclusion criteria were that the patients were aged <70 years, and that they had medial osteoarthritis or spontaneous osteonecrosis of the knee, correction angles < 10°, pre- and postoperative MRI T2 mapping, and follow-up until hardware removal. Patients were excluded if they had a Kellgren–Lawrence grade 4 of knee OA, had undergone preoperative MRI at another medical facility prior to referral to our hospital, or they had refused to undergo a postoperative MRI. For preoperative planning, full-length standing anteroposterior radiographs of the legs were plotted using digital planning software (Ortho Planner Pro version 3.5.5; Toyo Technica, Osaka, Japan). To achieve appropriate realignment of the lower limb and valgus correction, the target mechanical axis (MA) was set at 62.5% from the medial edge of the tibial plateau to its entire length (Fujisawa point). For patients with correction angles of <10°, OWHTO was the procedure of choice. For patients with correction angles of ≥10°, flexion contracture exceeding 10°, or anterior knee pain, closed wedge high tibial osteotomy was recommended.

### 2.2. Radiological Evaluation

**Figure 1:**
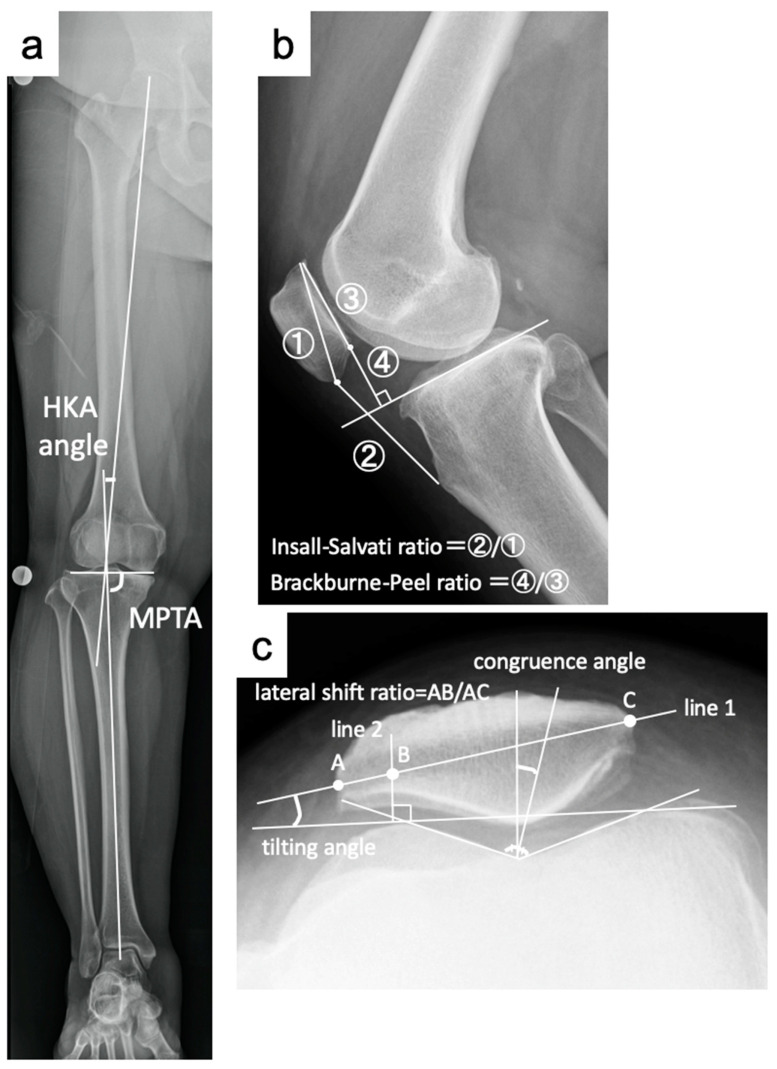
(**a**) Method used to measure the hip-knee-ankle angle (HKAA) and medial proximal tibial angle (MPTA). (**b**) Method used to measure the Insall–Salvati ratio (②/①) and Blackburne–Peel ratio (④/③). (**c**) Method used to measure the patellar tilt angle, lateral shift ratio and congruence angle. A: the intersection of line 1 and the lateral patella side. B: the intersection of line 1 and line 2. C: the intersection of line 1 and the medial patella side.

Preoperative and pre-hardware removal radiographic images of the knee joint were evaluated. The hip–knee–ankle angle (HKAA) and medial proximal tibial angle (MPTA) were measured using full-length standing images as described previously (Figure 1) [12,13]. Merchant imaging was employed to measure the patellar tilt angle (PTA), sulcus angle (SA), lateral shift ratio (LSR), and congruence angle (CA) to evaluate the PF joint with the knee in flexion at 45° [14,15]. The Insall–Salvati (IS) and Blackburne–Peel (BP) ratios were determined at 30° of knee flexion to assess patellar height [16,17]. The presence and severity of PF osteoarthritis were assessed using the Kellgren–Lawrence grading system [18].

### 2.3. MRI T2 Mapping

Magnetic resonance imaging (MRI) using a 1.5-tesla system (Brivo MR355 Inspire; GE Healthcare, Chicago, IL, USA) was performed on all knees before surgery and 6 months postoperatively. The imaging protocol included turbo spin echo (TSE), multi-echo time (TE), repetition time (TR) of 1200 ms, and TE of 7.5/15/22.5/30/37.5/45/52.5/60 ms. The protocol also included a field of view (FOV) of 160 mm, slice thickness of 3.0 mm, slice gap of 0.6 mm, 15 slices, 2 excitations, bandwidth of 31.25 kHz, scan time of 5 min and 22 s, and a 224 × 224 matrix. The T2 mapping images were calculated and generated from a series of images.

**Figure 2:**
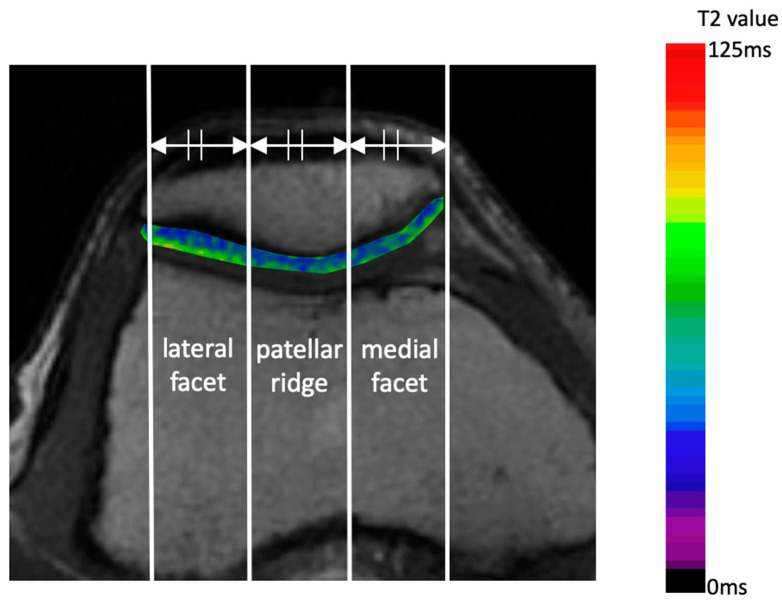
Axial image of the center of the patella of T2 mapping. The depth of the region of interest in the articular cartilage was set to encompass the superficial and intermediate layers.

The axial plane at the center of the patella was analyzed on T2 mapping images to evaluate the impact of OWHTO and LRR on the PF joint. The patellar width was divided into three equal parts, establishing three regions of interest (ROI): the medial facet, patellar ridge, and lateral facet (Figure 2).

The depth of each ROI was set to encompass the superficial and middle layers of the patellar cartilage, with a margin of a few pixels away from the articular cartilage surface to avoid partial volume effects. Pre- and postoperative T2 values were compared to determine the effect on cartilage degeneration. Mean T2 values were calculated using the AZE Virtual Place AVP-001A (Canon Medical Systems Corporation, Otawara, Japan) [19]. All measurements were conducted in a blinded, independent manner by two orthopedic surgeons with expertise in knee surgery: one with 20 years of experience and the other with 25 years of experience. The average of the calculated values was used.

### 2.4. Surgical Procedure

Prior to osteotomy, the International Cartilage Repair Society (ICRS) grades were determined using arthroscopy [20]. OWHTO was conducted according to previously described methodology [5]. During OWHTO, the osteotomy line was set 35 mm from the articular surface. Following biplane osteotomy, the medial side was opened and filled with artificial bone (Olympus Terumo Biomaterials Co., Tokyo, Japan). In the OWHTO + LRR group, LRR was performed within the joint using radiofrequency ablation (RFA) under arthroscopy. The lateral patellar retinaculum was dissected 1 cm from the lateral edge of the patella, distal to the anterolateral portal, and proximal to one finger width proximal to the patella. Following transection, the mobility of the patella was confirmed to have improved by manual lifting of the lateral side of the patella [21]. After surgery, the patient was placed on a partial weight-bearing regimen, starting with half of the recommended partial weight-bearing and progressing to two-thirds, contingent upon the patient’s pain tolerance. Full weight bearing was permitted 2 weeks post-surgery.

### 2.5. Statistical Analysis

Statistical analyses were performed using EZR (Saitama Medical Center, Jichi Medical University, Saitama, Japan) and a graphical user interface for R (R Foundation for Statistical Computing, version 2.13.0). Data were expressed as the mean ± SD and analyzed accordingly. Student’s t-test was used to analyze quantitative data, whereas Fisher’s exact test was used to analyze qualitative data. Statistical significance was set at *p* < 0.05. The total sample size (*n* = 34) provided >80% power to detect a difference in T2 values in the patellar ridge of the OWHTO group (α = 0.05; β = 0.20).

## 3. Results

Table 1 shows the pre- and post-surgery patient characteristics.

No significant differences were observed in patient backgrounds between the OWHTO and OWHTO + LRR groups. Furthermore, no significant differences were observed in the Knee Injury and Osteoarthritis Outcome Score subscale scores preoperatively and after hardware removal. In the radiological evaluation, both groups demonstrated a statistically significant increase in the HKAA and MPTA following surgery compared with the preoperative values (Table 2).

The BP ratio, which is an indicator of patellar height, declined in both groups. PTA, which serves as an indicator of patellar alignment, showed a notable decline in both groups. However, LSR and CA showed no discernible changes. No significant differences were observed between the two groups in the amount of change including in the ΔHKAA, ΔMPTA, ΔIS ratio, and ΔBP ratio in the radiographic evaluations before and after surgery (Table 3).

Furthermore, no significant differences in ΔLSR and ΔCA were observed between the two groups. However, the ΔPTA value for the OWHTO group (−2.9 ± 2.1) was significantly larger than that for the OWHTO + LRR group (−1.0 ± 2.6) (*p* < 0.01). Additionally, the ICRS grades of the patellar and femoral sides remained unchanged in both groups (Table 4).

**Figure 3:**
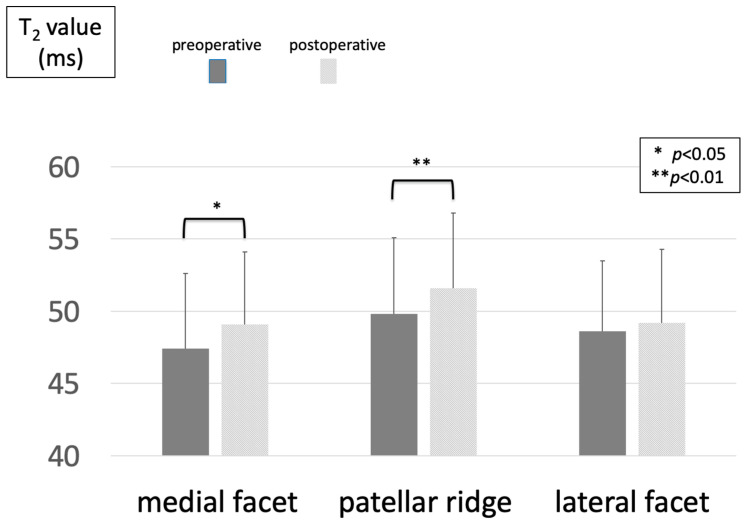
Changes in T_2_ values before and after OWHTO.

The mean T2 values in the OWHTO group exhibited a significant increase in both the medial facet and patellar ridge (medial facet: 47.4 ± 5.2 to 49.1 ± 5.0; patellar ridge: 49.8 ± 5.3 to 51.6 ± 5.2) (*p* < 0.01) (Figure 3).

**Figure 4:**
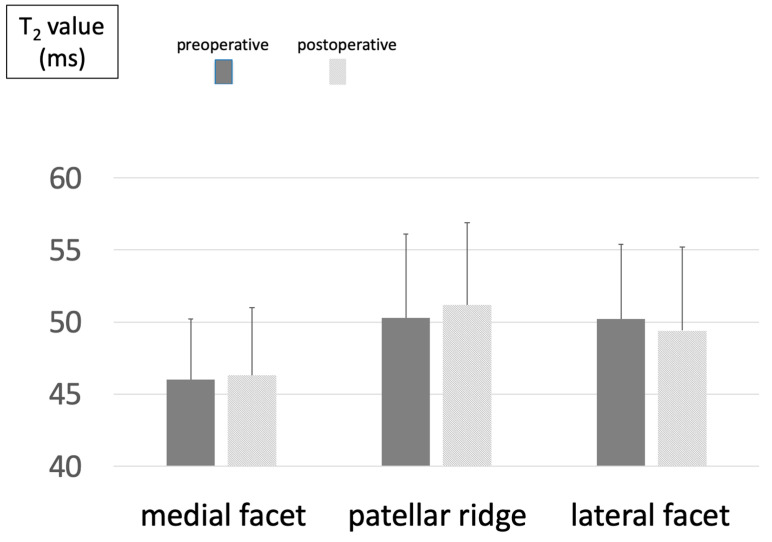
Changes in T_2_ values before and after OWHTO + LRR.

In contrast, no significant postoperative change was observed in any ROI in the OWHTO + LRR group (medial facet: 46.0 ± 4.2 to 46.3 ± 4.7; patellar ridge: 50.3 ± 5.8 to 51.2 ± 5.7) (Figure 4).

## 4. Discussion

The most significant outcome of this study was that, although the T2 values of the medial facet and patellar ridge had increased considerably in the OWHTO group at 6 months post-surgery, no analogous increase was observed in the OWHTO + LRR group. This suggests that the combination of LRR and OWHTO effectively mitigated the qualitative alterations in the articular cartilage of the PF joint in the early postoperative phase. One potential mechanism is that the combination with LRR may have suppressed the medial inclination of the patella following surgery.

The articular cartilage is composed of 70% water, 20% collagen, and 10% proteoglycans. Collagen fibers are arranged in a regular formation; this maintains the shape of the cartilage and limits the motility of the water molecules. T2 mapping is an MRI method used to qualitatively evaluate articular cartilage [22]. The alignment of the collagen and the water content of articular cartilage can be evaluated using T2 mapping. Normal T2 values range from 45.04 ms to 46.9 ms [23]. Early cartilage degeneration due to collagen damage and changes in collagen content or arrangement will increase the water mobility in the tissue, thus increasing the T2 value. Therefore, T2 mapping is a useful method of detecting early degeneration of articular cartilage.

The use of OWHTO has become widespread since the advent of locking plates, which permit early weight-bearing following surgery, and the relatively straightforward nature of the procedure. However, recent reports have indicated potential complications, including patella baja and degeneration of the articular cartilage of the PF joint, which may be evident during second-look surgery after OWHTO [4]. Many reports have demonstrated that no correlation exists between clinical scores and outcomes in the short-to medium-term. Goshima et al. observed that, with an average of 10.8 years follow-up after OWHTO, clinical outcomes were not affected in 94.7% of cases when total knee arthroplasty was used as the endpoint [24]. Nevertheless, conventional clinical scores may not be able to accurately differentiate between pain originating from the PF and that originating from the femorotibial joint. Additionally, the long-term outcomes of PF joint disorders after OWHTO remain inadequately evaluated. Consequently, OWHTO that preserves the PF joint is preferable. A significant complication associated with OWHTO is the potential for major correction, which can lead to PF joint disorders. In a study by Otakara et al., a change in the MPTA of ≥10° significantly increased the risk of damage to the PF joint [5]. Therefore, OWHTO is indicated when the correction is minor. In this study, the degeneration of the PF joint cartilage in the early stages after OWHTO was evaluated using the T2 mapping method. Even when the correction was <10°, qualitative changes occurred in the PF joint cartilage, in the medial facet and patellar ridge. Therefore, PF joint disorders may occur even with small corrections in OWHTO.

In contrast, LRR is primarily indicated when there is an issue with the PF joint. It has been used to treat conditions such as OA of the PF joint, patellar instability, and excessive lateral pressure syndrome [25,26,27,28]. In an in vitro study, Ostermeier et al. reported that LRR increases the medial tilt of the patella and decreases the pressure on its lateral facet [29]. The use of closed-wedge HTO in conjunction with LRR can effectively inhibit degeneration of the PF joint while simultaneously enhancing patellar alignment and clinical outcomes [30,31]. However, comparatively fewer studies have combined LRR with OWHTO. In a study employing plain radiographs, Li et al. reported that in OWHTO, the Kellgren–Lawrence grades of the PF joint progressed in approximately 2% of cases, whereas in the group with combined LRR, no cases progressed [32]. In this study, we used MRI to conduct a qualitative assessment of the PF joint cartilage in the early postoperative period in cases where OWHTO and LRR were performed in conjunction. In the OWHTO + LRR group, no statistically significant increase was observed in the T2 values of any of the ROIs on the patella. This indicates that qualitative alterations in the articular cartilage on the patellar aspect may have been averted by the LRR. Moreover, no significant difference was observed between the postoperative KOOS of the two groups. Because this was a relatively short-term evaluation, we believe that further long-term clinical evaluation is necessary.

Furthermore, in addition to patellar baja, sagittal alignment of the patella has been identified as a potential contributing factor to the effect of OWHTO on the PF joint. In a biomechanical study using cadaver knees, Gaasbeek et al. analyzed dynamic patellar tracking [33]. While no alteration in lateral patellar shift was observed resulting from OWHTO, a notable increase was observed in medial patellar tilt. We propose that OWHTO results in increased lateral tension of the patella, with medial tilt resulting from the lateral facet of the patella being pressed against the lateral wall of the groove. In addition, clinical studies have indicated that despite a lack of alteration in lateral patellar shift, a reduction in the lateral patellar tilt occurred following OWHTO [34,35]. In the current study, as in previous studies, LSR remained unchanged in the OWHTO group, whereas PTA exhibited a reduction. In a study examining patellar alignment using OWHTO combined with LRR, Murayama et al. reported a reduction in lateral patellar shift and lateral patellar tilt when OWHTO with a correction of ≥10 mm was combined with LRR [36]. Li et al. reported a reduction in lateral shift and lateral tilt when LRR was used in conjunction with OWHTO in patients presenting with anterior knee pain, osteoarthritis of the PF joint, cartilage damage of the PF joint, and excessive lateral pressure syndrome [32]. In this study, the combination of LRR and OWHTO, which corrected for 10 mm or less, did not result in a greater change in lateral shift than with OWHTO alone. However, the lateral tilt decreased. The decrease in lateral tilt was significantly smaller than that in the group that underwent OWHTO alone. Therefore, in this study, we hypothesized that although the use of LRR in combination with OWHTO increased the medial patellar tilt in sagittal alignment, the effect was less than that with OWHTO alone, which may have reduced the degeneration of the patellar articular cartilage. A potential reason for this discrepancy with previous studies is the differences in the indications for surgery.

This study had a few limitations. First, the OWHTO and OWHTO + LRR groups were not randomized. Second, it had a small sample size and retrospective design. Third, it was not possible to ascertain the impact of LRR in conjunction with OWHTO in patients in whom the PF joint exhibited a correction > 10°, as the procedure was performed using closed-wedge HTO in such instances. Fourth, macroscopic evaluation of the articular cartilage was conducted at the time of hardware removal, limiting the ability to assess long-term qualitative changes in the PF joint caused by LRR. A longer follow-up period is required to evaluate articular cartilage degeneration of the PF joint.

## 5. Conclusions

In this study, we employed MRI T2 mapping to qualitatively assess the articular cartilage of the PF joint in cases in which OWHTO was used as a standalone procedure or in conjunction with LRR during the early postoperative period. In patients in whom OWHTO was used alone, a significant increase in T2 values was observed at 6 months postoperatively for the medial facet and patellar ridge. In contrast, the use of LRR in combination with OWHTO suppressed the qualitative changes in the PF joint cartilage during the early postoperative period. The combination of LRR and OWHTO was effective in mitigating the impact on the PF joint by minimizing alterations in patellar alignment.

## Figures and Tables

**Table 1:** Patient characteristics.

	OWHTO Group	OWHTO + RL Group	*p*-Value
gender (male/female)	14/23	9/28	0.32
age at surgery, mean ± SD (years)	59.5 ± 10.5	59.3 ± 9.4	0.95
height (cm)	163.0 ± 8.5	162.5 ± 5.7	0.76
weight (kg)	64.8 ± 10.7	64.9 ± 13.5	0.98
body mass index (kg/m^2^)	24.3 ± 2.7	24.5 ± 4.4	0.84
preoperative KOOS			
ADL	74.0 ± 16.5	77.6 ± 17.0	0.35
pain	60.9 ± 15.1	64.1 ± 20.7	0.45
QOL	38.3 ± 17.7	42.2 ± 20.2	0.38
sports	44.1 ± 23.0	52.0 ± 25.0	0.16
symptom	64.4 ± 17.6	70.7 ± 17.0	0.12
postoperative KOOS			
ADL	90.7 ± 9.8	91.4 ± 11.6	0.80
pain	82.6 ± 13.4	84.8 ± 15.2	0.55
QOL	69.7 ± 21.1	71.8 ± 22.6	0.70
sports	70.8 ± 21.4	71.3 ± 22.5	0.93
symptom	82.8 ± 12.7	86.1 ± 14.1	0.32
opening width (mm)	8.3 ± 2.2	8.2 ± 2.1	0.82

**Table 2:** Pre- and postoperative radiological evaluation.

	OWHTO Group			OWHTO + RL Group		
	Pre	Post	*p* Value	Pre	Post	*p* Value
HKA angle (°)	−5.0 ± 2.6	3.4 ± 1.6	<0.001 *	−4.3 ± 2.0	4.0 ± 1.6	<0.001 *
MPTA (°)	84.1 ± 1.8	91.6 ± 2.0	<0.001 *	84.6 ± 2.4	92.3 ± 2.6	<0.001 *
IS ratio	1.11 ± 0.13	1.11 ± 0.14	0.53	1.15 ± 0.17	1.15 ± 0.17	0.95
BP ratio	0.85 ± 0.08	0.71 ± 0.10	<0.001 *	0.88 ± 0.11	0.75 ± 0.10	<0.001 *
tilting angle (°)	8.6 ± 3.7	5.7 ± 3.6	<0.001 *	7.9 ± 3.6	6.9 ± 4.2	0.02 *
lateral shift ratio	12.6 ± 4.8	11.0 ± 5.5	0.07	12.5 ± 5.2	12.1 ± 6.0	0.69
congruence angle (°)	−5.8 ± 10.6	−6.3 ± 10.4	0.55	−7.2 ± 9.1	−7.4 ± 7.5	0.85

* *p* < 0.05.

**Table 3:** Changes in alignment and patellar parameters in the two groups.

	OWHTO Group	OWHTO + RL Group	*p*-Value
ΔHKA (°)	8.4 ± 2.2	8.3 ± 2.1	0.85
ΔMPTA (°)	7.6 ± 2.2	7.7 ± 1.9	0.77
ΔIS ratio	0.0 ± 0.1	0.0 ± 0.1	0.63
ΔBP ratio	−0.15 ± 0.11	−0.13 ± 0.10	0.43
Δtilting angle	−2.9 ± 2.1	−1.0 ± 2.6	<0.01 *
Δlateral shift ratio	−1.7 ± 5.4	−0.3 ± 5.1	0.28
Δcongruence angle	−0.5 ± 5.5	−0.2 ± 5.3	0.76

* *p* < 0.05.

**Table 4:** Pre- and postoperative patellofemoral joint cartilage changes.

		OWHTO Group	OWHTO + RL Group	*p*-Value
ICRS grade (0/1/2/3/4)				
patella	pre	2/22/10/3/0	0/25/12/0/0	0.15
	post	1/21/12/2/1	0/24/13/0/0	0.37
femur	pre	0/17/15/5/0	0/10/23/4/0	0.16
	post	0/12/16/9/0	0/13/21/3/0	0.16

## Data Availability

The original contributions presented in this study are included in the article. Further inquiries can be directed to the corresponding author.

## Abbreviations

The following abbreviations are used in this manuscript:

OWHTO	Open-wedge high tibial osteotomy
LRR	Lateral retinacular release
MRI	Magnetic resonance imaging
KOA	Osteoarthritis of the knee
PF	Patellofemoral
MA	Mechanical axis
HKAA	Hip–knee–ankle angle
MPTA	Medial proximal tibial angle
PTA	Patellar tilt angle
SA	Sulcus angle
LSR	Lateral shift ratio
CA	Congruence angle
IS	Insall–Salvati
BP	Blackburn–Peel
MPTA	Medial proximal tibial angle
TSE	Turbo spin echo
TE	Multi-echo time
TR	Repetition time
FOV	Field of view
RFA	Radiofrequency ablation
